# PKPD modeling of the inoculum effect of combined ceftazidime/avibactam and colistin against KPC-3 *Klebsiella pneumoniae* isolate

**DOI:** 10.1128/aac.01797-24

**Published:** 2025-04-14

**Authors:** Romain Aubry, Julien M. Buyck, Alexia Chauzy, Laure Prouvensier, Jean-Winoc Decousser, Patrice Nordmann, Sebastian G. Wicha, Sandrine Marchand, Nicolas Grégoire

**Affiliations:** 1 INSERM U1070 PHAR2, Université de Poitiers 27077, Poitiers, Nouvelle-Aquitaine, France; 2 Laboratoire de Toxicologie et de Pharmacocinétique, CHU de Poitiers 36655, Poitiers, Nouvelle-Aquitaine, France; 3 Department of Bacteriology and Infection Control, University Hospital Henri Mondor, Assistance Publique-Hôpitaux de Paris, Créteil, France; 4 EA 7380 Dynamyc Université Paris-Est Créteil (UPEC), Ecole nationale vétérinaire d'Alfort (EnvA), Faculté de Médecine de Créteil, Créteil, France; 5 Medical and Molecular Microbiology, Faculty of Science and Medicine, University of Fribourg 98839, Fribourg, Switzerland; 6 Swiss National Reference Center for Emerging Antibiotic Resistance (NARA), University of Fribourg 27211, Fribourg, Switzerland; 7 Institute for Microbiology, University of Lausanne and University Hospital Centre, Lausanne, Switzerland; 8 Department of Clinical Pharmacy, Institute of Pharmacy, University of Hamburg 276709, Hamburg, Germany; Providence Portland Medical Center, Portland, Oregon, USA

**Keywords:** antimicrobial combination, PKPD modeling, ceftazidime/avibactam, colistin, inoculum effect

## Abstract

The inoculum effect (IE) characterizes a decrease in the antimicrobial effect of antibiotics with increasing inoculum. To face antimicrobial resistance, antibiotic combinations are progressively used. In this context, the effect of combination may be affected by IE, especially drugs for which an IE has been described. The objective was to characterize the IE of a carbapenemase (KPC-3) *Klebsiella pneumoniae* isolate on the combination of ceftazidime/avibactam (CZA) and colistin (CST). *In vitro* time-kill curves with single and combined drugs were performed at four different inocula. The IE of each drug was described using pharmacokinetic/pharmacodynamic modeling, and interactions on IE were investigated with the general pharmacodynamic interaction model when drugs were combined. The IE was assessed by evaluating the significance of the parameters associated with the IE model compared to the no IE model and by comparing the CFU counts over time predicted with the IE model vs the no IE model. Rapid bacterial killing was observed at 10^4^ CFU/mL. For both 5·10^5^ and 10^7^ CFU/mL inocula, initial decays followed by re-growth were observed with drugs alone, while the combination prevented the emergence of resistance. Eradication was never achieved at 10^8^ CFU/mL. The IE was best modeled as a reduction of CZA maximum bactericidal effect and as an increase in CST EC_50_ with increasing inoculum. However, no interaction between IEs was significant, meaning that CST did not modify the IE of CZA and inversely. IE may be important at least as demonstrated by *in vitro* antibiotic combination studies.

## INTRODUCTION

The inoculum effect (IE) characterizes a decrease in the antimicrobial effect of antibiotics with increasing inoculum concentration (1). This complex phenomenon has been extensively investigated *in vitro* and has been reported to be dependent on the antibiotic–strain pair (2). However, antibiotic combinations are being increasingly used to face antimicrobial resistance (3). In this context, IE may impact the effect of combined drugs, especially those for which an IE has been described in monotherapy. Previous *in vitro* studies have reported a minor to moderate IE of ceftazidime/avibactam (CZA) against Enterobacterales (4–6), while a significant IE of colistin (CST) has been demonstrated on many occasions in Enterobacterales (7–9).

Several mechanisms have been proposed to explain the IE, including an unbalanced ratio between the free antibiotic concentration and the bacterial density (10), an enhanced production of β-lactamases (1, 2, 11), an increased subpopulation of resistant bacteria (12, 13), a change in the physiological state of the bacteria (13), a decrease in the expression of penicillin-binding proteins (PBPs) (14), a rapid development of biofilms (15), and quorum sensing (12). Because the European Committee on Antimicrobial Susceptibility Testing and the Clinical and Laboratory Standards Institute guidelines usually rely on a standard 5·10^5^ CFU/mL inoculum (16, 17), the IE is not taken into consideration for determining clinical breakpoints. Therefore, antibiotic regimens used to treat infections with elevated bacterial burden, such as intra-abdominal or respiratory infections (1), may become ineffective and favor antimicrobial resistance.

Traditionally, the IE was defined as an eightfold or higher minimum inhibitory concentration (MIC) increase at high inoculum (≥10^7^ CFU/mL) compared to the standard inoculum size (18). Although practical, the MIC only provides evidence of a change in the drug susceptibility at a given time when the bacterial inoculum increases. The IE was also investigated based on more informative time-kill curves (TKCs), and the relationship between the inoculum size and the antibiotic efficacy was further characterized using pharmacokinetic/pharmacodynamic (PKPD) modeling (12, 13, 19). This quantitative approach was used to predict the time course of the bacterial density, depending on drug concentrations, time, and starting inoculum. The IE of antibiotic combination has only been studied once, reporting CFU counts at several time points (8). However, to our knowledge, no article has reported a PKPD modeling approach for quantification of IE on antibiotics used in combination and, in particular, how IEs found when antibiotics were alone applied in combination.

The objective of this study was to characterize the IE of a KPC-3 *Klebsiella pneumoniae* isolate on the combination of CZA and CST, taken as an example of a multidrugresistant strain expressing a carbapenemase. To investigate the IE, TKCs were performed at several inoculum sizes, and a PKPD model was built to quantify this effect. Then, to evaluate its impact, two methodologies were applied, one evaluating the significance of the parameters associated with the IE and one comparing the CFU counts over time.

## MATERIALS AND METHODS

### Antibiotics

Ceftazidime and avibactam were purchased separately from MedChemExpress (NJ, USA). Colistin was purchased from Sigma Aldrich (Saint-Quentin-Fallavier, France). Stock solutions of antibiotics (10,240 mg/L) were prepared in sterile water, stored at −80°C, and diluted in Mueller–Hinton broth (MHB) II, cation-adjusted on the day of the experiment to achieve the desired concentrations.

### Strains

A clinical KPC-3 *K. pneumoniae* isolate (NARA864), obtained from the National Reference Center for Emerging Antibiotic Resistance (Fribourg, Switzerland) and susceptible to CZA (MIC = 2 mg/L with fixed avibactam concentration of 4 mg/L) and CST (MIC = 0.25 mg/L), was used. This strain had already been used to study the CZA + CST interaction in a previous work (20).

### Static time-kill experiments

*In vitro* time-kill experiments included a growth control, single-drug experiments with ceftazidime ranging from 0.5 to 8.0 mg/L with twofold serial dilution (with fixed avibactam concentration of 4 mg/L) or CST alone, ranging from 0.06 to 8.0 mg/L. The CZA + CST combination was tested at several concentrations ranging from 0.5 + 0.06 mg/L to 4.0 + 0.5 mg/L, respectively. Bacterial inocula of 10^4^, 5·10^5^, 10^7^, and 10^8^ CFU/mL were investigated, and all the TK experiments were performed independently from the same bacterial stock.

One microliter of a highly concentrated frozen bacterial suspension was taken from a −80°C storage and incubated overnight in 10 mL of MHB at 35°C with constant shaking (130 rpm) using an orbital shaker. A 200 µL volume of this bacterial suspension was diluted in 10 mL of MHB and pre-incubated (2 h, 35°C, 130 rpm) to reach the exponential growth phase. The optical density at 600 nm was measured with a spectrophotometer and adjusted between 0.08 and 0.11 (0.5 McFarland), corresponding to a 10^8^ CFU/mL bacterial density in the exponential growth phase. Then, this bacterial suspension was either adequately diluted to achieve a final inoculum of 10^4^, 5·10^5^, or 10^7^ CFU/mL, or it was centrifuged at 2,500 RCF for 6 min, and the pellet was re-suspended in MHB to concentrate the suspension and achieve a final inoculum of 10^8^ CFU/mL. The final preparation (10 mL total volume) containing the antibiotics at targeted concentrations was incubated at 35°C with constant shaking (130 rpm) for 30 h. Samples were taken at 0, 2, 4, 8, 24, and 30 h, serially diluted in NaCl 0.9% and plated on Mueller–Hinton agar using an easySpiral automatic plater (Interscience, France). Plates were incubated for 24 h at 35°C, and CFUs were enumerated using a SCAN 300 colony counter (Interscience). The lower limit of quantification was 200 CFU/mL. Each experimental condition was tested one to four times.

### Model development with or without inoculum effect

**Fig 1:**
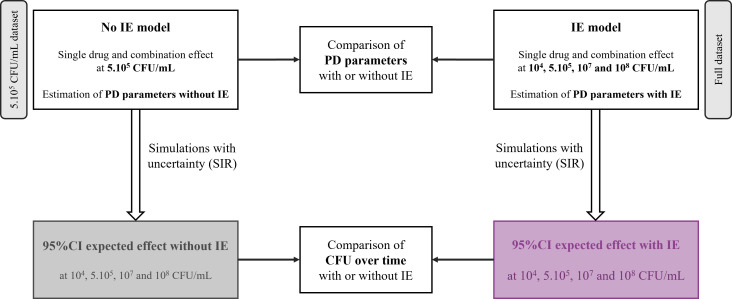
Flowchart for building models with or without IE and for evaluating inoculum effect. IE, Inoculum Effect; SIR, Sampling Importance Re-sampling (22); PD, pharmacodynamic.

The workflow for building models with or without IE and for evaluating IE is summarized in Fig. 1. Data sets were prepared using R software (version 3.6.2), and the parameters of the model were estimated using NONMEM software (version 7.4) with the Laplacian algorithm. Data below the limit of quantification were handled using Beal’s M3 method (21). Evaluation and selection of the model were based on objective function value (OFV), goodness-of-fit plots, and accuracy of parameter estimates (relative standard errors). Model building is further detailed in Supplemental Text S1.

**Fig 2:**
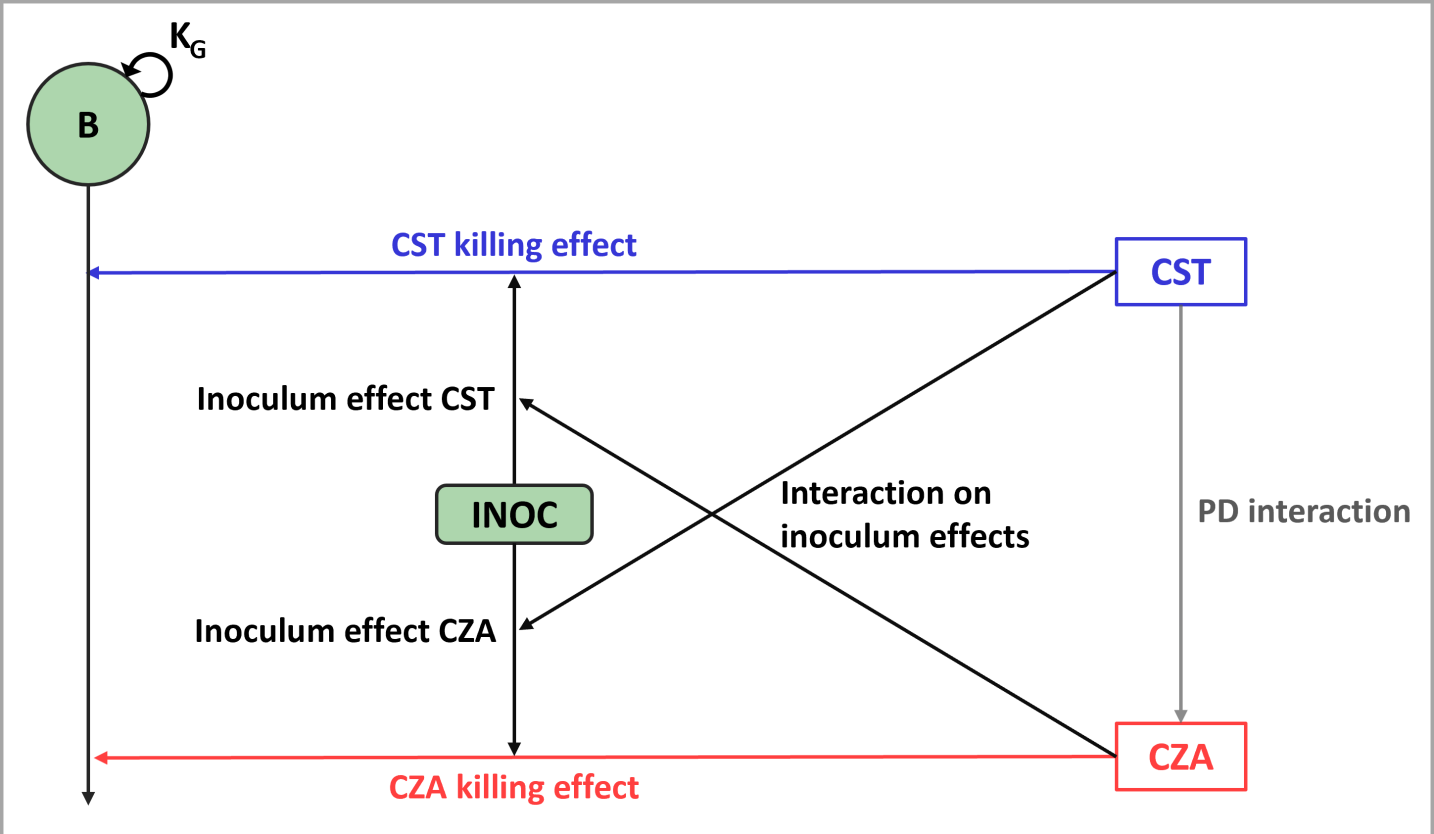
Development of the PKPD model with inoculum effect. *B*, total bacterial population; CST, colistin; CZA, ceftazidime/avibactam; INOC, starting inoculum; *K*_*G*_, growth rate. The General PharmacoDynamic Interaction (GPDI) model (23) was used to investigate the possibility that in combination, each antibiotic modified the IE of the other drug.

In the first step of the analysis, single-drug and combination effects at 5·10^5^ CFU/mL standard inoculum were described by using a previously developed PKPD model (20), re-estimating the parameters on the basis of experimental data from the present study. This model is referred to as the “no IE model.” Using this model, the expected bactericidal curves (with their 95% confidence intervals [CIs]) were predicted at 10^4^, 10^7^, and 10^8^ CFU/mL inocula, corresponding to the expected effects without IE. In the second step of the analysis, the no IE model was adapted to take into account the IE and fitted to the full data set including all tested inocula. The IE of each drug, as well as the possibility that in combination each antibiotic modified the IE of the other drug, was also tested (Fig. 2). To describe the IE of each drug, multiple functions, including linear, power, and (sigmoidal) maximum bactericidal effect (Emax) equations, were tested, resulting in a change of Emax, EC_50_, or Kon of the corresponding drug. The change of IE due to the combination with the other drug was investigated using the general pharmacodynamic interaction model (22). The selection of the model that best described the observed data, socalled “IE model,” was based on the difference in OFV between models. The NONMEM control stream of the IE model is provided in Supplemental Code S1.

### Evaluation of the inoculum effect

The IE was assessed in two ways, firstly, by the significance of the parameters associated with the IE model compared to the no IE model, and secondly, by the differences in CFU counts over time between the profiles predicted with the IE model and those predicted with the no IE model. The comparison of predicted effects over time according to whether experiments were performed with a single inoculum or with several inocula was performed by superimposing the 95% CIs predicted from the IE model and from the no IE model. The 95% CIs were constructed using a sampling importance re-sampling (SIR) procedure, applied with the SIR tool of PsN (version 5.3.0), as described in Supplemental Text S1.

### PKPD simulations at varying clinical concentrations

In order to evaluate the impact of the inoculum effect in the case of varying concentrations, the CZA + CST combination effect was simulated with the no IE model and with the IE model at concentrations mimicking the concentrations obtained for a typical patient after administration of standard dosage regimens. PKPD simulations were performed using the PK model of ceftazidime from Sy et al. (23) and the PK model of CST from Kristoffersson et al. (24), assuming a typical patient (weight = 74.4 kg, creatinine clearance = 120 mL/min). The clinical dosing regimen of ceftazidime 2 g was administered every 8 h as a 2 h intravenous infusion, and a loading dose of 9 million units (MIU) of colistin methanesulfonate sodium (CMS) followed by maintenance CMS doses (4.5 MIU) every 12 h was administered as a 30 min intravenous infusion. Unbound fractions of CAZ and CST in plasma were assumed to be 85% (25) and 34% (26), respectively. The PK of avibactam was not simulated since our *in vitro* data could not support the activity of avibactam at varying concentrations, and it was assumed that avibactam concentration was sufficient to inhibit β-lactamases and had no bactericidal effect on its own.

## RESULTS

### Models with or without inoculum effect

**Fig 3:**
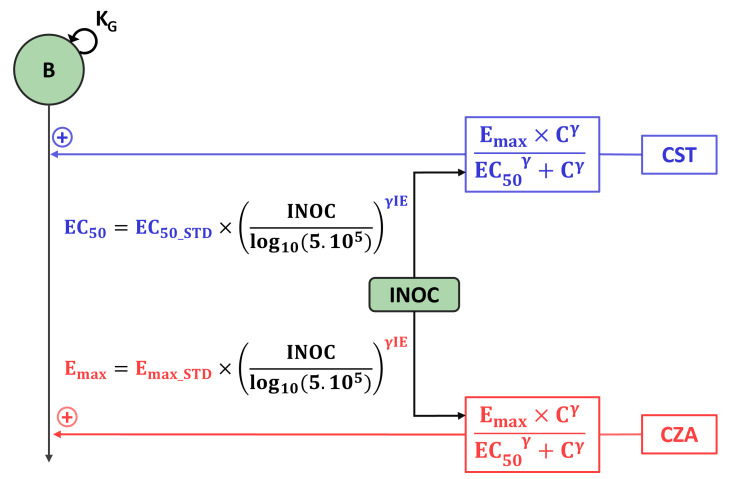
Diagram of the IE model. *B*, total bacterial population; *K*_*G*_, growth rate; INOC, starting inoculum; *C*, drug concentration; Emax, maximum bactericidal effect; EC_50_, concentration for which the effect is 50% of Emax; *γ*, power parameter for drug effect; γIE, power parameter for inoculum effect. Parameters in blue refer to CST and parameters in red refer to CZA. Adaptive resistance and PD interaction between CST and CZA were omitted to ease reading.

**Fig 4:**
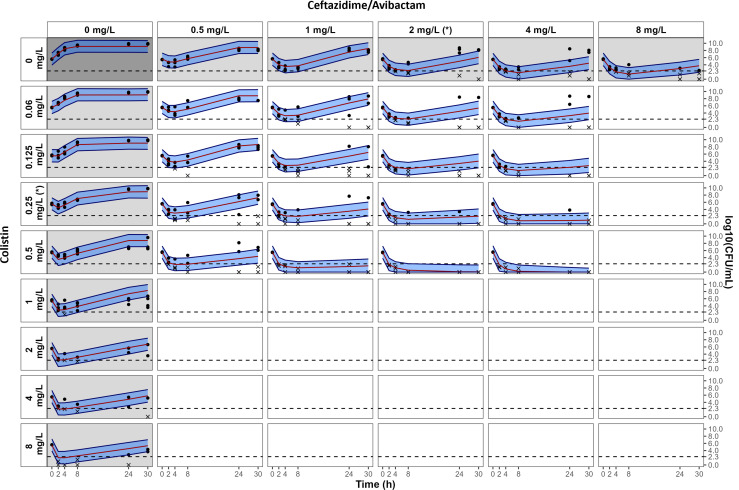
Visual predictive checks of the IE model at 5·10^5^ CFU/mL. Gray and white panels are associated with single-drug and combination experiments, respectively. Measured CFUs are represented by dots. For graphical representation, data below the limit of quantification are represented by an “x” at their measured values. The median percentile from simulations with the IE model is represented by a red line, and the 80% prediction interval between the 10th and 90th percentiles is represented by the blue-shaded areas. The limit of quantification is represented by the dashed line at 2.3 log_10_ CFU/mL. MICs of CZA and CST are indicated by (*). Avibactam concentration was fixed at 4 mg/L.

**Fig 5:**
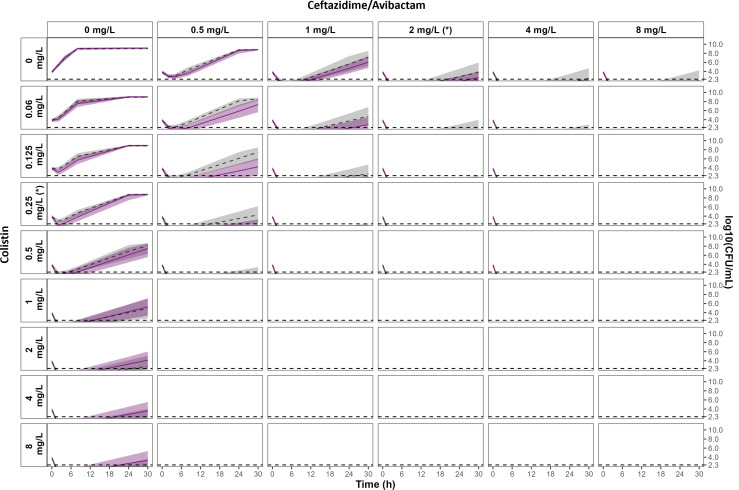
Comparison of the 95% CIs of the effect predicted with the no IE (gray area) and with the IE (purple area) models at 10^4^ CFU/mL. The 95% CI of the effect predicted with the no IE model, obtained by SIR, is represented by gray areas, and the corresponding median percentile is represented by the dashed line. The 95% CI of the effect predicted with the IE model is represented by light purple areas, and the median percentile is represented by the solid line. Both 95% CIs were systematically overlapping, indicating that CFU counts over time were not significantly affected by the IE. The limit of quantification is represented by the horizontal dashed line at 2.3 log_10_ CFU/mL. MICs of CZA and CST are indicated by (*).

**Fig 6:**
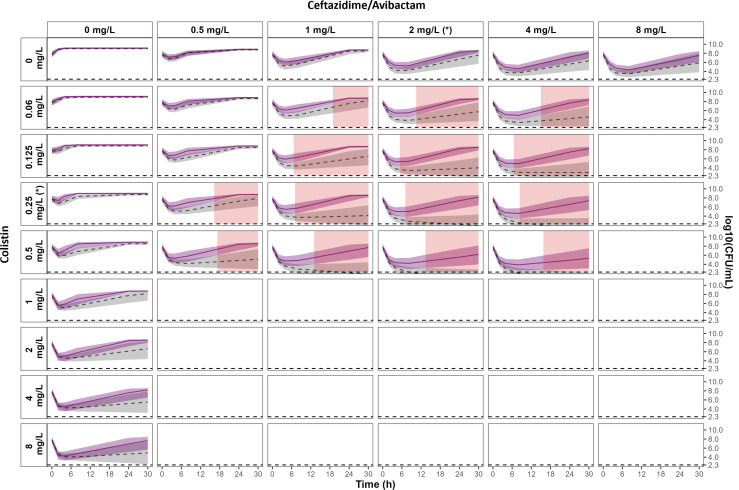
Comparison of the 95% CIs of the effect predicted with the no IE (gray area) and with the IE (purple area) models at 10^8^ CFU/mL. The 95% CI of the effect predicted with the no IE model, obtained by SIR, is represented by gray areas, and the corresponding median percentile is represented by the dashed line. The 95% CI of the effect predicted with the IE model is represented by light purple areas, and the median percentile is represented by the solid line. Statistically significant areas of decreased drug effect due to inoculum effect are highlighted in red (non-overlapping 95% CIs*)*. The limit of quantification is represented by the horizontal dashed line at 2.3 log_10_ CFU/mL. MICs of CZA and CST are indicated by (*).

The no IE model, developed using only data with an inoculum of 5·10^5^ CFU/mL, fitted the data correctly. The visual predictive checks (VPCs) of the no IE model and parameter estimates are presented in Fig. S1 and Table S1, respectively. A schematic representation of the IE model, developed using data at all inocula, is shown in Fig. 3, and parameter estimates are given in Table S2. The complete PD model structure is schematized in Fig. S2. The VPCs presented in Fig. 4 and Fig. S3 to S5 show that for the different inocula, the observed data were correctly fitted by the IE model. The comparisons between the 95% CIs of the effect predicted with the no IE model and with the IE model at the four inoculum sizes are shown in Fig. 5 and 6; Fig. S6 and S7.

Measured inocula were in accordance with the targeted values, yet being slightly lower with median observations of 10^3.85^ CFU/mL (relative bias vs targeted inoculum = −29%), 10^5.53^ CFU/mL (−32%), 10^6.97^ CFU/mL (−7%), and 10^7.87^ CFU/mL (−26%), respectively. At 10^4^ CFU/mL starting inoculum, a rapid killing of the bacteria was achieved with drugs alone and in combination, except at the lowest concentrations (Fig. S3). For both 5·10^5^ and 10^7^ CFU/mL inoculum, an initial decay followed by bacterial re-growth was observed with CZA and CST alone, while the combination in the range of 1× to 2× the MIC prevented the emergence of resistance (Fig. 4; Fig. S4, respectively). Bacterial eradication was never achieved for the high inoculum (10^8^ CFU/mL), even at the highest drug concentrations (Fig. S5).

### Evaluation of the inoculum effect

The IE was best modeled as a statistically significant reduction of the Emax of CZA and as an increase of the EC_50_ of CST with increasing inoculum according to equation 1. Emax__CZA_ varied from 6.01 h^−1^ (95% CI 5.59–6.42 h^−1^) to 5.21 h^−1^ (95% CI 4.84–5.54 h^−1^), i.e., a mean reduction of 13%, and the EC50__CST_ from 0.15 mg/L (95% CI 0.13–0.18 mg/L) to 0.44 mg/L (95% CI 0.36–0.52 mg/L); i.e., a mean increase of 193% when the inoculum increased from 10^4^ to 10^8^ CFU/mL, respectively.


equation 1 $$\theta_{DRUG}=\theta_{DRUG\_STD}\times {\left(\frac{INOC}{\;\log_{10}\left(5.10^{5}\right)\;}\right)}^{\gamma IE_{DRUG}},$$

where $\theta_{DRUG}$ is the parameter value of the corresponding drug (i.e., Emax__CZA_ [h^−1^] or EC_50_CST_ [mg/L]) at the estimated inoculum size; $\theta_{DRUG\_STD}$ is the typical parameter value of the corresponding drug (Emax__CZA_STD_ or EC_50_CST_STD_) estimated at the standard 5·10^5^ CFU/mL starting inoculum; INOC (log_10_ CFU/mL) is the estimated inoculum size for each experiment; and $\gamma IE_{DRUG}$ is the estimated coefficient to describe the IE of the corresponding drug. A positive value of $\gamma IE_{DRUG}$ leads to an increase in $\theta_{DRUG}$ with increasing inoculum, while a negative value results in a decrease in $\theta_{DRUG}$ with increasing inoculum. The higher the parameter, the higher the change in $\theta_{DRUG}$. No significant interaction was found between the IEs of CZA and CST, meaning that the presence of CST did not modify the IE of CZA, and the presence of CZA did not modify the IE of CST. The estimated parameters for IEs are presented in Table 1.

**TABLE 1:** Parameter estimates of the inoculum effect model^*d*^

Description	Parameter	Estimate (RSE %)
Inoculum effect of CZA^*a*^		
Maximum kill rate constant of CZA (h^−1^) at 5·10^5^ CFU/mL	Emax__CZA_STD_	5.59 (3)
Inoculum effect on Emax__CZA_	γIE_CZA_	−0.21 (13)
Inoculum effect of CST^*b*^		
CST concentration required to achieve 50% of Emax__CST_ (mg/L) at 5·10^5^ CFU/mL	EC_50_CST_STD_	0.26 (9)
Inoculum effect on EC50_CST_		1.53 (10)
Interactions on inoculum effects^*c*^		
Interaction of CZA on the inoculum effect of CST	E_CZA__IE_CST_	0
Interaction of CST on the inoculum effect of CZA	E_CST__IE_CZA_	0

^*a*^ $\begin{array}{rl} & {Emax}_{CZA}={Emax}_{CZA}{}_{STD}\times {\left(\frac{INOC}{\log_{10}\left(5.10^{5}\right)}\right)}^{\gamma IECZA} \end{array}$

^*b*^ $\begin{array}{rl} & EC500_{CST}=EC50_{CST\text{ STD }}\times {\left(\frac{INOG}{\log_{10}\left(5.10^{5}\right)}\right)}^{\gamma IEE_{CST}} \end{array}$

^*c*^ None of the interactions on inoculum effects were significant.

^*d*^ CST, colistin; CZA, ceftazidime/avibactam (fixed to 4 mg/L); IE, inoculum effect; RSE %, relative standard error (%) estimated using sampling importance re-sampling.

The results with a single drug at all inoculum sizes showed that the 95% CI of the effect predicted with the IE model overlapped with the 95% CI of the effect predicted with the no IE model, indicating that bacterial counts over time were not significantly affected by the IE of CZA alone (first row on Fig. 5 and 6; Fig. S6 and S7) or CST alone (first column on Fig. 5 and 6; Fig. S6 and S7).

In combination, the results at the highest inoculum (10^8^ CFU/mL) showed a significant reduction of the effect of the CZA + CST combination (highlighted in red in Fig. 6) compared to what was predicted from the no IE model. Indeed, the combination of both drugs was expected to achieve a killing effect, whereas bacterial re-growth was observed at this high inoculum.

### PKPD simulations at varying clinical concentrations

**Fig 7:**
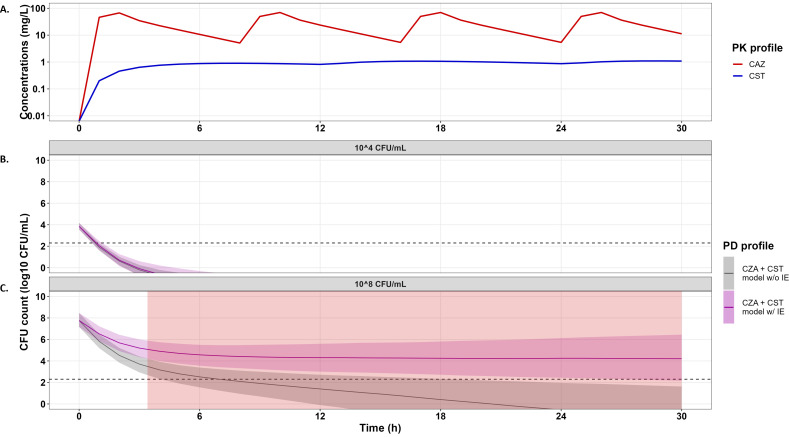
PKPD simulation in combination with the No IE (gray area) and with the IE (purple area) models, at 10^4^ CFU/mL (**B**) and 10^8^ CFU/mL inoculum (**C**) for typical free CAZ and CST concentrations (**A**). Free PK profiles were simulated in typical patients after standard doses of ceftazidime (2 g every 8 h as 2 h infusion) and colistin (9 MIU CMS + 4.5 MIU CMS every 12 h as 30 min infusion). Statistically significant areas of decreased drug effect due to inoculum effect are highlighted in red (non-overlapping 95% CIs).

Simulation results at 10^4^ CFU/mL (Fig. 7B), 5·10^5^ CFU/mL (Fig. S8B), and 10^7^ CFU/mL (Fig. S8C) showed that the combination of usual dosing regimens of CZA and CST should result in a fast killing of the bacteria. In contrast, at 10^8^ CFU/mL inoculum, the combination of CZA and CST with standard dosage regimens should not eradicate the bacteria (purple area, Fig. 7C). It should be noted that the model predicted that the IE was significant at these variable concentrations, and if the simulations had been carried out without taking IE into account, eradication of the bacteria would have been predicted (gray area, Fig. 7C).

## DISCUSSION

In the literature, two PKPD modeling approaches have been used to describe the IE. The first one assumes a static IE over time using an additional mathematical function to modify the antimicrobial effect solely based on the starting inoculum. For instance, in the study of Bhagunde et al. (15), the effective drug concentration, which determined the kill rate of the antimicrobial agent, was assumed to be inversely dependent on the initial bacterial burden, using a sigmoidal model. Similarly, Akrong et al. (19) characterized the IE of *Acinetobacter baumannii* on polymyxin B as an increase in the EC_50_ at higher inoculum, using a power function. In contrast, the second approach assumes a dynamic process where the IE changes according to the time course of the bacterial density using the mechanism-based model. Bulitta et al. (12, 27) suggested that all viable bacteria synthesized and released freely diffusible signal molecules that slowed down the bacterial replication and could cause a phenotypic change in bacterial cells. Nielsen et al. (13) characterized the IE of *Escherichia coli* strains on ciprofloxacin using a model structure with two different bacterial states: an actively growing drug-susceptible state (S) and a non-growing drug-insusceptible resting state (R). In their model, the transfer from bacterial state S to R was assumed to be proportional to the bacterial density over time and thus reduced the drug effect at a higher inoculum. Since our experimental data did not support any particular mechanism, we characterized the IE using an empirical and static PKPD model similar to that of Akrong et al. (19).

In our model, the IE of CZA was best described as a decrease in the Emax parameter, meaning that even if the antibiotic concentration was increased, the maximum effect observed at a lower inoculum could not be reached. In the case of CZA, the decrease of Emax could be explained by a reduction in the expression or an alteration of specific PBPs (1, 14). Indeed, at a high inoculum, the stationary phase is rapidly reached, and bacterial cell division is reduced, resulting in a reduction in the quantity of target for CZA. A reduction in the acylation of PBPs at a high inoculum may also be responsible for a reduction in the target expression and may explain the decrease in Emax (28). On the other hand, CST interacts with the lipopolysaccharides of the outer membrane of the bacteria (29). Therefore, at a high inoculum, the total number of targets is increased, and a higher CST concentration is required to achieve the same effect. Accordingly, the IE was best fitted by an increase in CST EC_50_. These hypotheses are in agreement with the mathematical models describing our observations but should be verified experimentally. In addition, the potential interaction of CST on the IE of CZA (and conversely) was investigated, but none of these significantly improved the PD model, therefore suggesting that both IEs were independent when CZA and CST were used in combination.

Traditionally, the IE is assessed by comparing the MIC values obtained at the standard inoculum (5·10^5^ CFU/mL) and at an elevated starting inoculum. The effects are therefore compared at a given time. TKCs enable the evaluation of the impact of the IE over time using PKPD models, particularly with regard to the emergence of resistance. In this situation, the significance of the IE is based on the improvement of the likelihood of the PKPD model when adding an inoculum effect. In accordance with previous studies reporting IE against CZA or CST, the magnitude of the IE of CST was higher compared to CZA. However, interpreting the impact of a change in a PD parameter (Emax of CZA and EC_50_ of CST in this study) may be complicated, while the CFU profiles over time can highlight how this change impacts the effect of single and combined drugs. The superimposition on the same figure of the typical profile estimated from a single inoculum and that estimated from several inocula makes it possible to visualize the importance of the inoculum effect. In this way, we can assess the error that would have been made if we had extrapolated the estimated efficacy from a single inoculum to situations where the initial inoculum was different. Parameter uncertainties were estimated in order to build the 95% CI of the effect predicted with the IE model and with the no IE model, but these confidence intervals were constructed from different data sets, including more data for the inoculum effect. The comparison between 95% CIs was therefore not a statistical test to compare the two models, IE vs no IE, but allowed us to compare the results obtained with experiments carried out with a single inoculum with the results obtained with several inocula, i.e., with more experimental data. Overall, the results showed that the IE did not significantly affect the CFU profiles except for the CZA + CST combination at 10^8^ CFU/mL, resulting in faster bacterial re-growth at concentrations close to the MICs (red areas in Fig. 6). Finally, CFU profiles were simulated at varying concentrations typically observed in patients receiving usual dosing regimens of CZA and CST. These simulations suggested that in a clinical setting, the CZA + CST combination should eradicate bacteria at the inoculum lower than 10^7^ CFU/mL (Fig. 7B; Fig. S8) but not in the case of higher inoculum (Fig. 7C). Yet, these results should be confirmed with further experiments, including *in vivo* infection models.

We acknowledge that TKCs were performed using a fixed avibactam concentration as done by EUCAST (4 mg/L). In our PD model, it was assumed that this concentration was sufficient to inhibit β-lactamases but not to induce bacterial killing or potentiate ceftazidime effect as reported previously (30). However, experiments performed in the *in vitro* hollow-fiber infection model showed that low avibactam exposures were associated with the selection of resistant mutants (31). Thus, the hypotheses we have made concerning the effect of avibactam need to be verified, and the re-growth observed at the highest inoculum could be explained by insufficient inhibition of the β-lactamases. Since our *in vitro* data could not support the activity of avibactam at varying concentrations, the PK of avibactam was not considered in the simulations. Hence, bacterial profiles simulated with a single drug and in combination must be considered with caution regarding model assumptions. In addition, the choice of simulating free concentrations of CZA and CST was intended to illustrate the inoculum effect at clinically relevant concentrations, but the results obtained are not intended to be predictive of clinical efficacy and must be considered with the inherent limitations of *in vitro* experiments. Similarly, the choice of using a CZA + CST combination with a CZA-susceptible strain of *K. pneumoniae* for these experiments was purely for illustrative purposes and is in no way a recommendation for clinical practice. Finally, for this analysis, we considered bacterial counts as continuous variables. It would have been more rigorous to consider them as count data, but this is rarely done in practice, and the methods for estimating uncertainties would have been different. From a methodological point of view, it would be interesting if the method we used for continuous data could be extended to count data.

To conclude, this PKPD modeling approach of *in vitro* TKCs allowed the characterization of the inoculum effect of a KPC-3 *K. pneumoniae* on antibiotic combination. CFU profiles over time were not significantly affected by the inoculum effect when CZA or CST was used alone. However, the effect of the CZA + CST combination was significantly reduced at an elevated 10^8^ CFU/mL inoculum. In addition, according to the model, the combination of usual dosing regimens of CZA and CST should be effective for the treatment of infections with low inoculum sizes but not with elevated bacterial burden. Overall, these results suggest that the inoculum effect may be important at least as demonstrated by *in vitro* antibiotic combination studies.

## SUPPLEMENTAL MATERIAL

Supplemental material aac.01797-24-s0001.docx Tables S1 and S2, Figures S1 to S8, Text S1, and Code S1.

ASM does not own the copyrights to Supplemental Material that may be linked to, or accessed through, an article. The authors have granted ASM a non-exclusive, world-wide license to publish the Supplemental Material files. Please contact the corresponding author directly for reuse.
