# Supplementary material for: PKPD modeling of the inoculum effect of combined ceftazidime/avibactam and colistin against KPC-3 Klebsiella pneumoniae isolate
Source: Antimicrob Agents Chemother. 2025 Apr 14;69(5):e01797-24. doi: 10.1128/aac.01797-24 (PMC12057351; doi:10.1128/aac.01797-24)
Supplement: Supplemental material — Tables S1 and S2, Figures S1 to S8, Text S1, and Code S1. [file aac.01797-24-s0001.docx]

**Table S1. Parameter estimates of the No IE model at 5.10^5^ CFU/mL**

| **Description** | **Parameter** | **Estimate (RSE%)** |
| --- | --- | --- |
| Estimated inoculum size at time 0 (log_10_CFU/mL) | INOC | 5.54 (fixed) |
| Apparent growth rate constant (h^-1^) | K_G_ | 1.76 (8%) |
| Maximal bacterial population supported by the system (log_10_CFU/mL) | Bmax | 9.03 (1%) |
| Maximum kill rate constant of CZA (h^-1^) | Emax__CZA_ | 7.53 (3%) |
| CZA concentration required to achieve 50% of Emax__CZA_ (mg/L) | EC_50_CZA_ | 0.46 (7%) |
| Sigmoïdicity parameter for CZA effect | γ_CZA_ | 1.66 (10%) |
| Rate constant for development of adaptive resistance to CZA (h^-1^) | K_ON_CZA_ | 0.49 (4%) |
| Rate constant for reversal of adaptive resistance (h^-1^) | K_OFF_ | 0 (fixed) |
| Maximal reduction of Emax__CZA_ when bacteria are in the adapted state (%) | ARmax__CZA_ | 80.0 (3%) |
| Maximum kill rate constant of CST (h^-1^) | Emax__CST_ | 8.94 (fixed) |
| CST concentration required to achieve 50% of Emax__CST_ (mg/L) | EC_50_CST_ | 0.23 (13%) |
| Sigmoïdicity parameter for CST effect | γ_CST_ | 1.11 (5%) |
| Rate constant for development of adaptive resistance to CST (h^-1^) | K_ON_CST_ | 0.77 (5%) |
| Maximal reduction of Emax__CST_ when bacteria are in the adapted state (%) | ARmax__CST_ | 81.0 (2%) |
| Drug responsible for the PD interaction | Perpetrator | CST |
| Maximal fractional change of Emax__CZA_ due to CST (%) | INT_Emax | -60.0 (23%) |
| Concentration of CST required to achieve 50% of INT_Emax (mg/L) | EC_50_INT_Emax_ | 0.38 (fixed) |
| Sigmoïdicity parameter for the combination effect | γ_INT_Emax_ | 1 (fixed) |
| Inter-experimental variability on inoculum size (%) | IEV_INOC | 9.40 (33%) |
| Additive residual error (log_10_CFU/mL) | RES_ADD | 2.06 (2%) |

CST: colistin; CZA: ceftazidime/avibactam (with avibactam fixed to 4 mg/L). RSE%, Relative Standard Error (%) estimated using Sampling Importance Resampling.

**Table S2. Parameter estimates of the IE model**

| **Description** | **Parameter** | **Estimate (RSE%)** |
| --- | --- | --- |
| Estimated inoculum size at time 0 (log_10_CFU/mL) | INOC* | 3.86 (fixed) |
|  |  | 5.54 (fixed) |
|  |  | 6.65 (fixed) |
|  |  | 7.76 (fixed) |
| Apparent growth rate constant (h^-1^) | K_G_ | 1.77 (6%) |
| Maximal bacterial population supported by the system (log_10_CFU/mL) | Bmax | 9.14 (1%) |
| **Maximum kill rate constant of CZA (h^-1^)** | **Emax__CZA_** | **5.59 (3%)** |
| CZA concentration required to achieve 50% of Emax__CZA_ (mg/L) | EC_50_CZA_ | 0.40 (6%) |
| Sigmoïdicity parameter for CZA effect | γ_CZA_ | 1.26 (8%) |
| Rate constant for development of adaptive resistance to CZA (h^-1^) | K_ON_CZA_ | 0.31 (7%) |
| Rate constant for reversal of adaptive resistance (h^-1^) | K_OFF_ | 0 (fixed) |
| Maximal reduction of Emax__CZA_ when bacteria are in the adapted state (%) | ARmax__CZA_ | 72.9 (2%) |
| **Inoculum effect on Emax__CZA_** | **γ_IE_CZA_** | **-0.21 (13%)** |
| Maximum kill rate constant of CST (h^-1^) | Emax__CST_ | 13.79 (11%) |
| **CST concentration required to achieve 50% of Emax__CST_ (mg/L)** | **EC_50_CST_** | **0.26 (9%)** |
| Sigmoïdicity parameter for CST effect | γ_CST_ | 1.21 (4%) |
| Rate constant for development of adaptive resistance to CST (h^-1^) | K_ON_CST_ | 1.28 (14%) |
| Maximal reduction of Emax__CST_ when bacteria are in the adapted state (%) | ARmax__CST_ | 89.1 (1%) |
| **Inoculum effect on EC_50_CST_** | **γ_IE_CST_** | **1.53 (10%)** |
| Drug responsible for the PD interaction | Perpetrator | CST |
| Maximal fractional change of Emax__CZA_ due to CST (%) | INT_Emax | -63.0 (11%) |
| Concentration of CST required to achieve 50% of INT_Emax (mg/L) | EC_50_INT_Emax_ | 0.30 (fixed) |
| Sigmoïdicity parameter for the combination effect | γ_INT_Emax_ | 1 (fixed) |
| **Effect of CZA on the inoculum effect of CST** | **E_CZA__IE_CST_** | **0 (NS)** |
| **Effect of CST on the inoculum effect of CZA** | **E_CST__IE_CZA_** | **0 (NS)** |
| Inter-experimental variability on inoculum size (%) | IEV_INOC | 4.30 (fixed) |
| Additive residual error (log_10_CFU/mL) | RES_ADD | 1.73 (4%) |

CST: colistin; CZA: ceftazidime/avibactam (with avibactam fixed to 4 mg/L); RSE%: Relative Standard Error (%) estimated using Sampling Importance Resampling; NS: Not significative.
* Inoculum and inter-experimental variability were estimated based on CFU measured at T0 only.

**
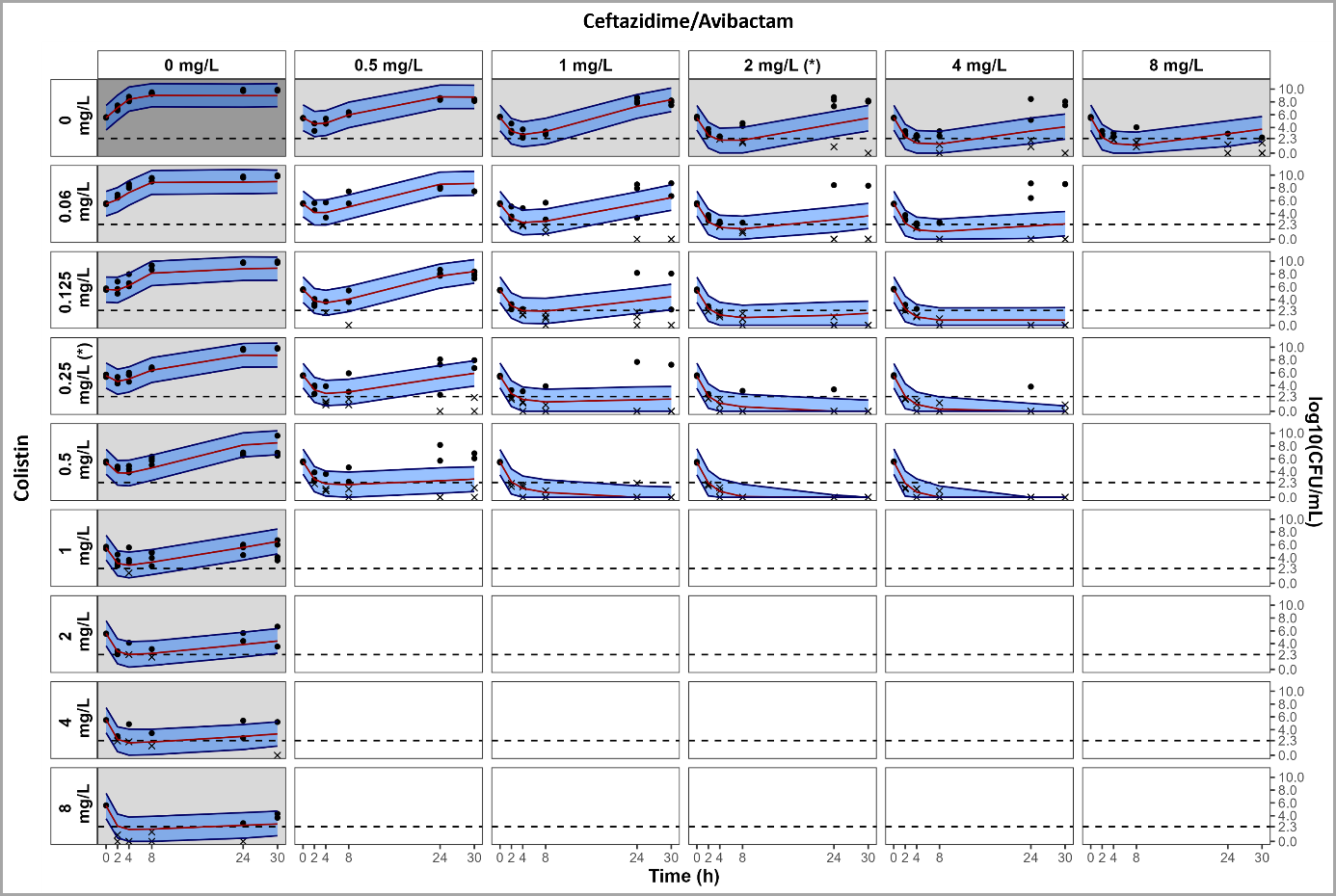
**

**Figure S1. Visual Predictive Checks of the No IE model at 5.10^5^ CFU/mL**

Grey and white panels are associated to single drug and combination experiments, respectively. Measured CFU are represented by dots. For graphical representation, data below limit of quantification are represented by cross at their measured values. Median percentile from simulations with the observed interaction model is represented by red line and the 80% prediction interval between 10th and 90th percentiles is represented by the blue shaded areas. Limit of quantification is represented by the dashed line at 2.3 log_10_CFU/mL. MICs of CZA and CST are indicated by (*). Avibactam concentration was fixed at 4 mg/L.

**
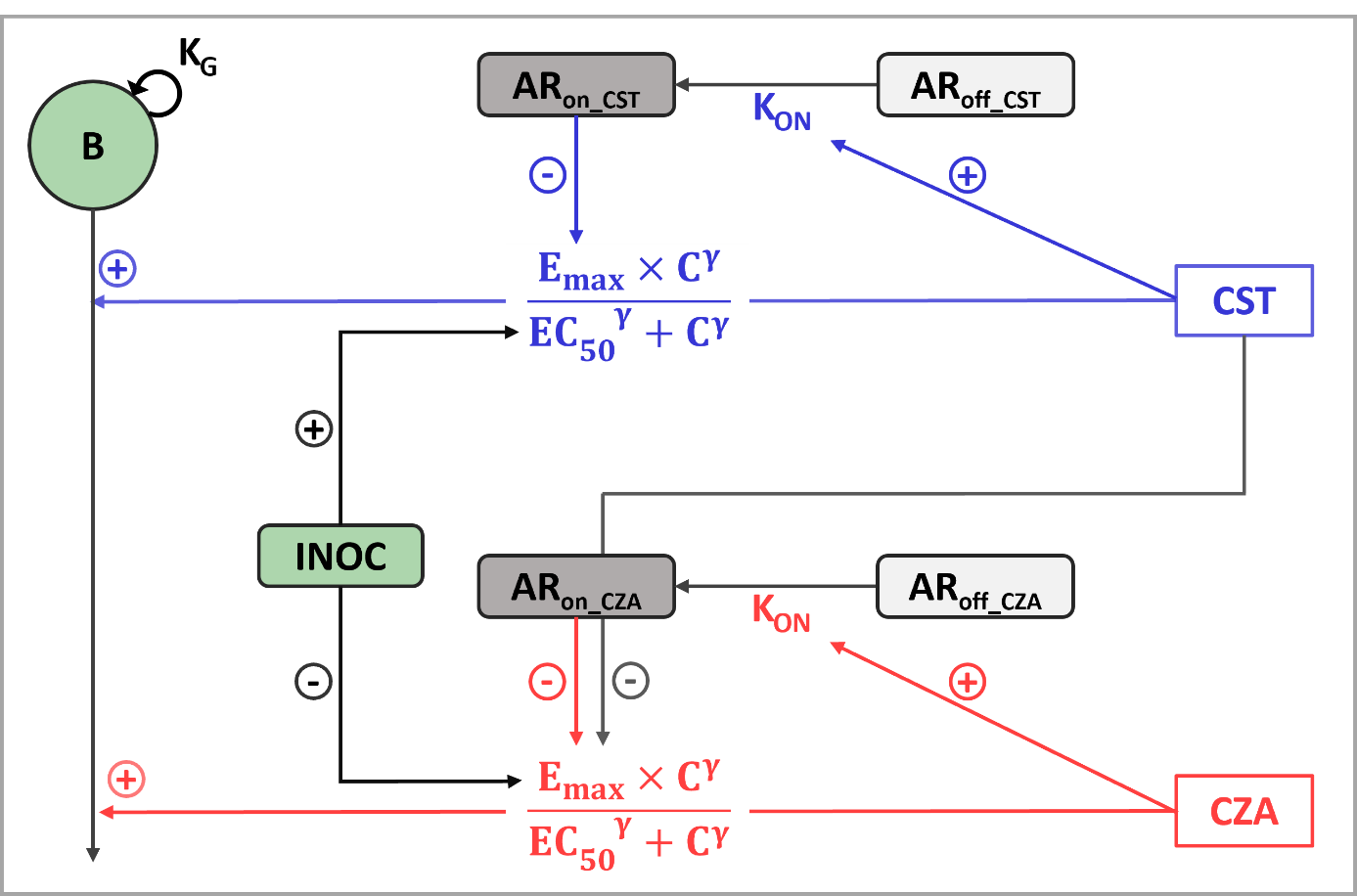
**

**Figure S2. Schematic diagram of the complete PD model structure**

B, total bacterial population; K_G_, growth rate; INOC, starting inoculum; AR_off_ and AR_on_, non-adapted and adapted state of the bacteria, respectively; K_on_, adaptation rate constant; Emax, maximum bactericidal effect; C, drug concentration; EC_50_, concentration for which effect is 50% of Emax; γ, power parameter for drug effect; parameters in blue refer to CST and parameters in red refer to CZA; the grey arrow represents the reduction of Emax_CZA_ due to CST using the GPDI model; the blacks arrows represent the inoculum effect.

**
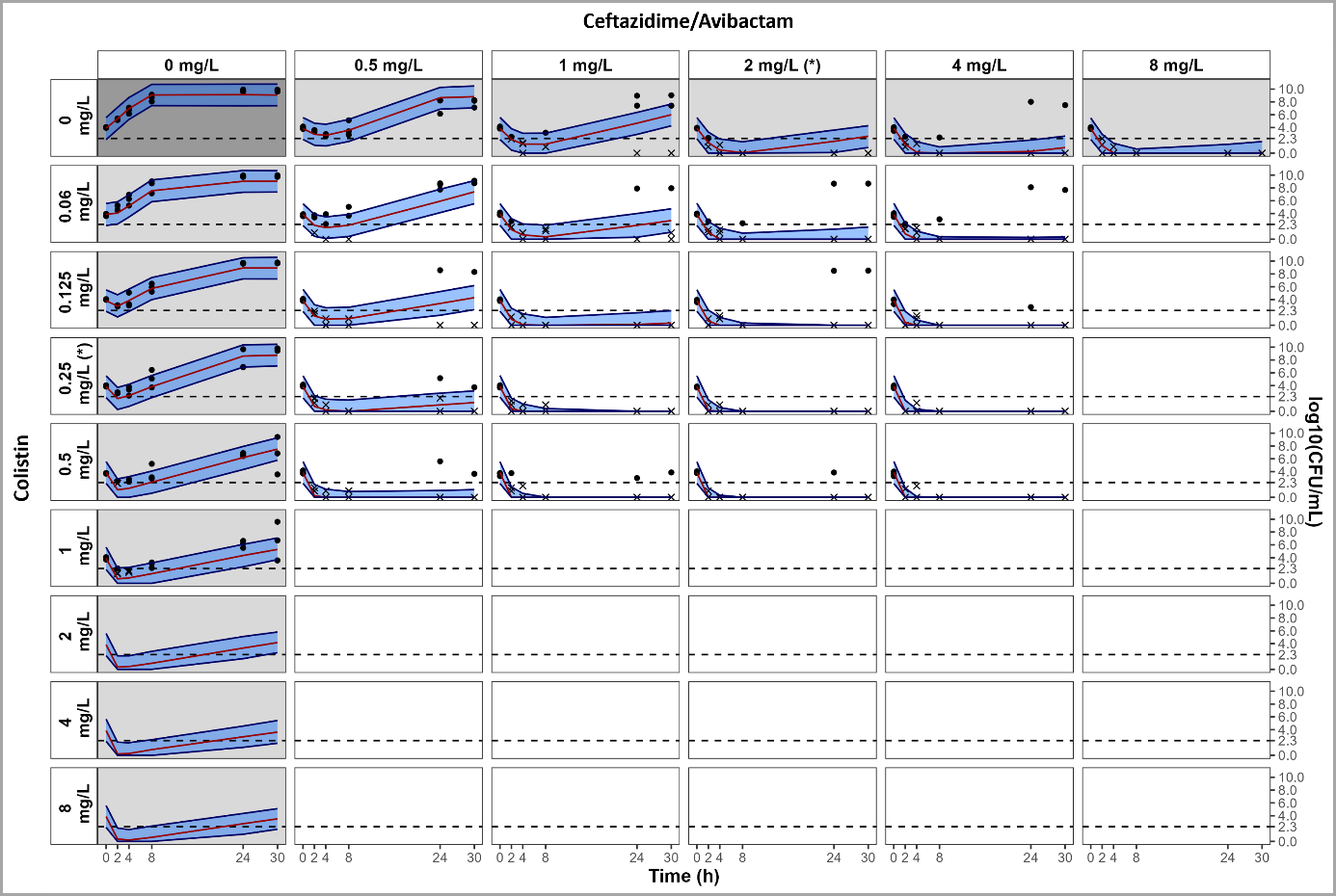
 Figure S3. Visual Predictive Checks of the IE model at 10^4^ CFU/mL**

Grey and white panels are associated to single drug and combination experiments, respectively. Measured CFU are represented by dots. For graphical representation, data below limit of quantification are represented by cross at their measured values. Median percentile from simulations with the observed interaction model is represented by red line and the 80% prediction interval between 10th and 90th percentiles is represented by the blue shaded areas. Limit of quantification is represented by the dashed line at 2.3 log_10_CFU/mL. MICs of CZA and CST are indicated by (*). Avibactam concentration was fixed at 4 mg/L.

**
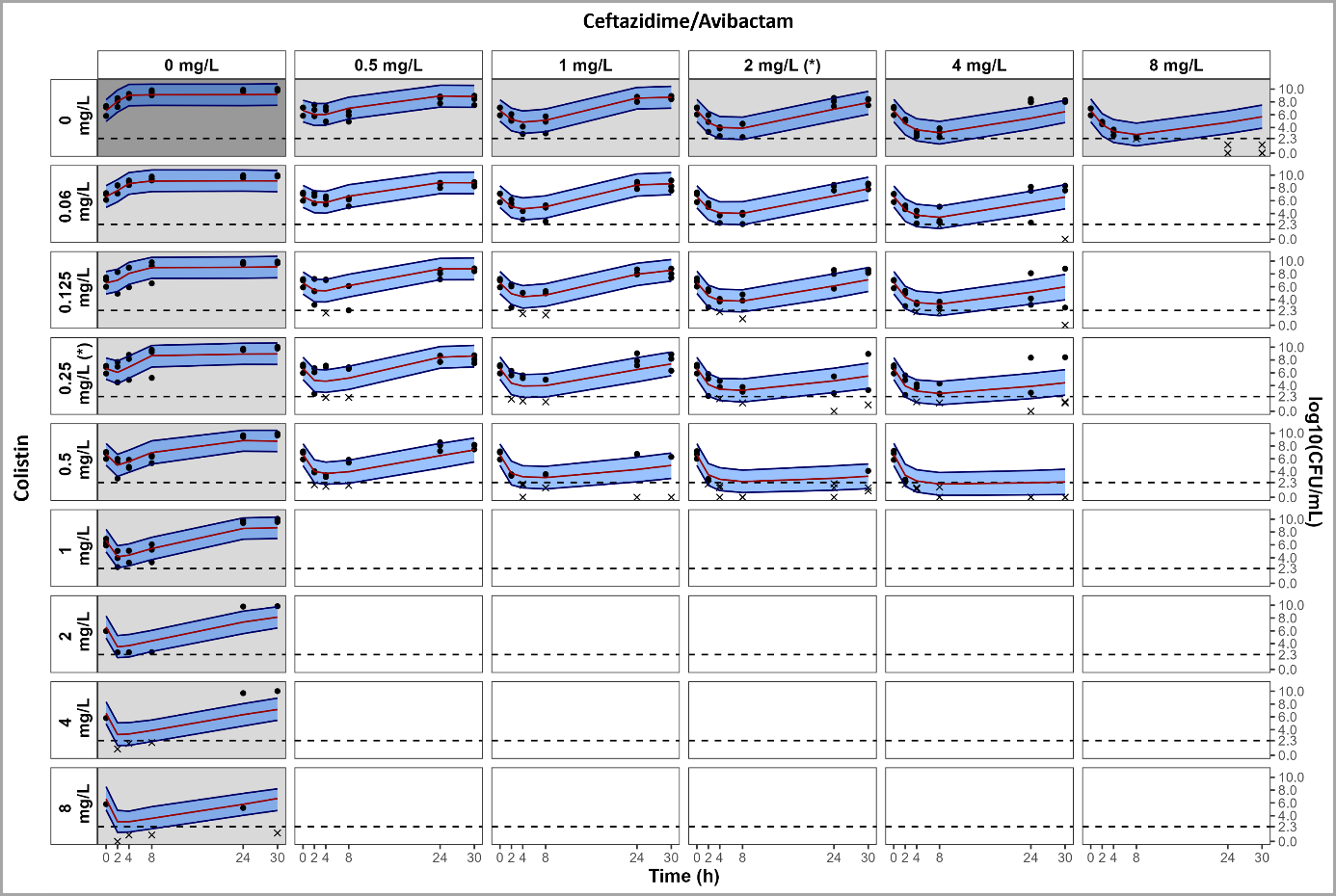
**

**Figure S4. Visual Predictive Checks of the IE model at 10^7^ CFU/mL**

Grey and white panels are associated to single drug and combination experiments, respectively. Measured CFU are represented by dots. For graphical representation, data below limit of quantification are represented by cross at their measured values. Median percentile from simulations with the observed interaction model is represented by red line and the 80% prediction interval between 10^th^ and 90^th^ percentiles is represented by the blue shaded areas. Limit of quantification is represented by the dashed line at 2.3 log_10_CFU/mL. MICs of CZA and CST are indicated by (*). Avibactam concentration was fixed at 4 mg/L.

**
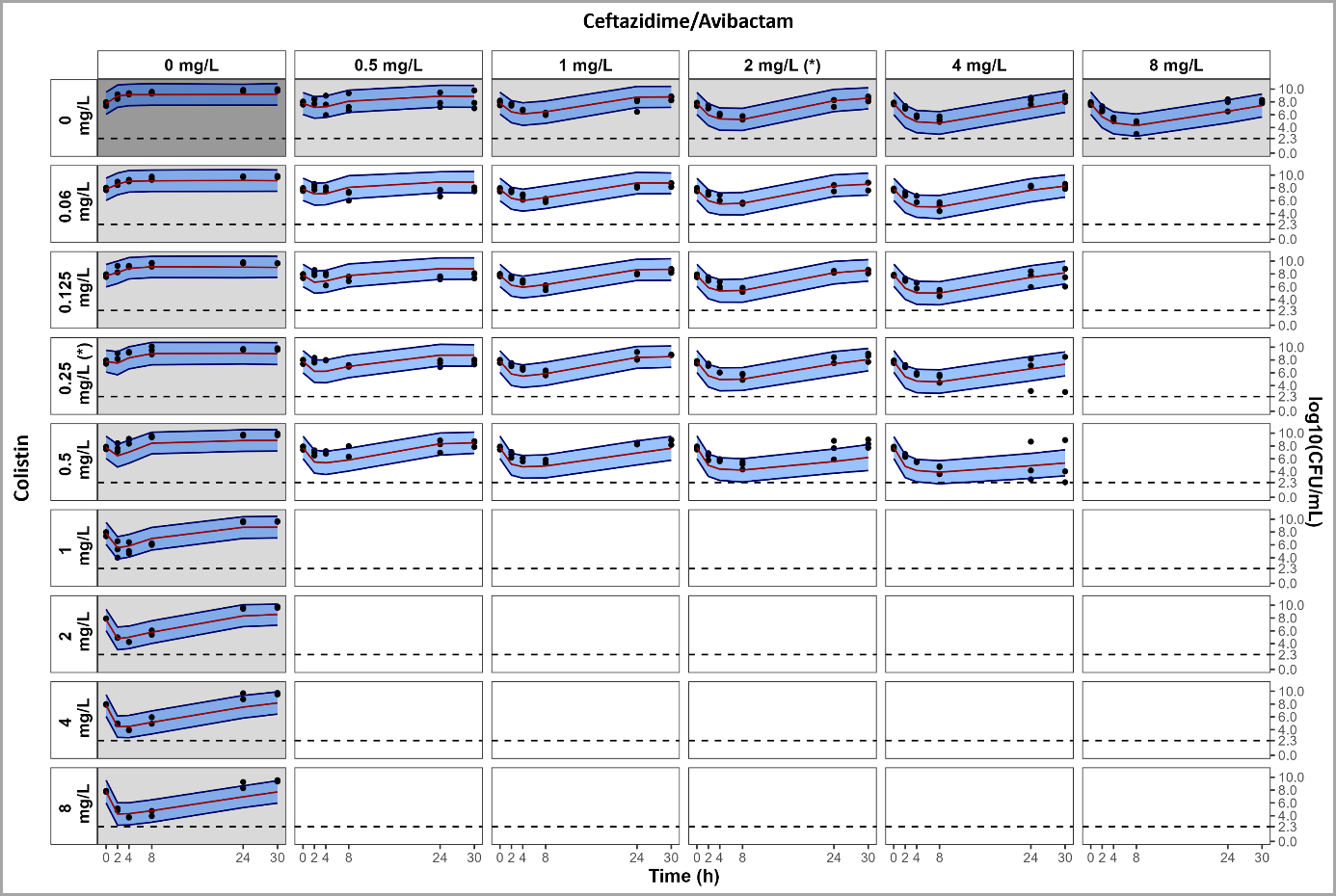
Figure S5. Visual Predictive Checks of the IE model at 10^8^ CFU/mL**

Grey and white panels are associated to single drug and combination experiments, respectively. Measured CFU are represented by dots. For graphical representation, data below limit of quantification are represented by cross at their measured values. Median percentile from simulations with the observed interaction model is represented by red line and the 80% prediction interval between 10^th^ and 90^th^ percentiles is represented by the blue shaded areas. Limit of quantification is represented by the dashed line at 2.3 log_10_CFU/mL. MICs of CZA and CST are indicated by (*). Avibactam concentration was fixed at 4 mg/L.

**
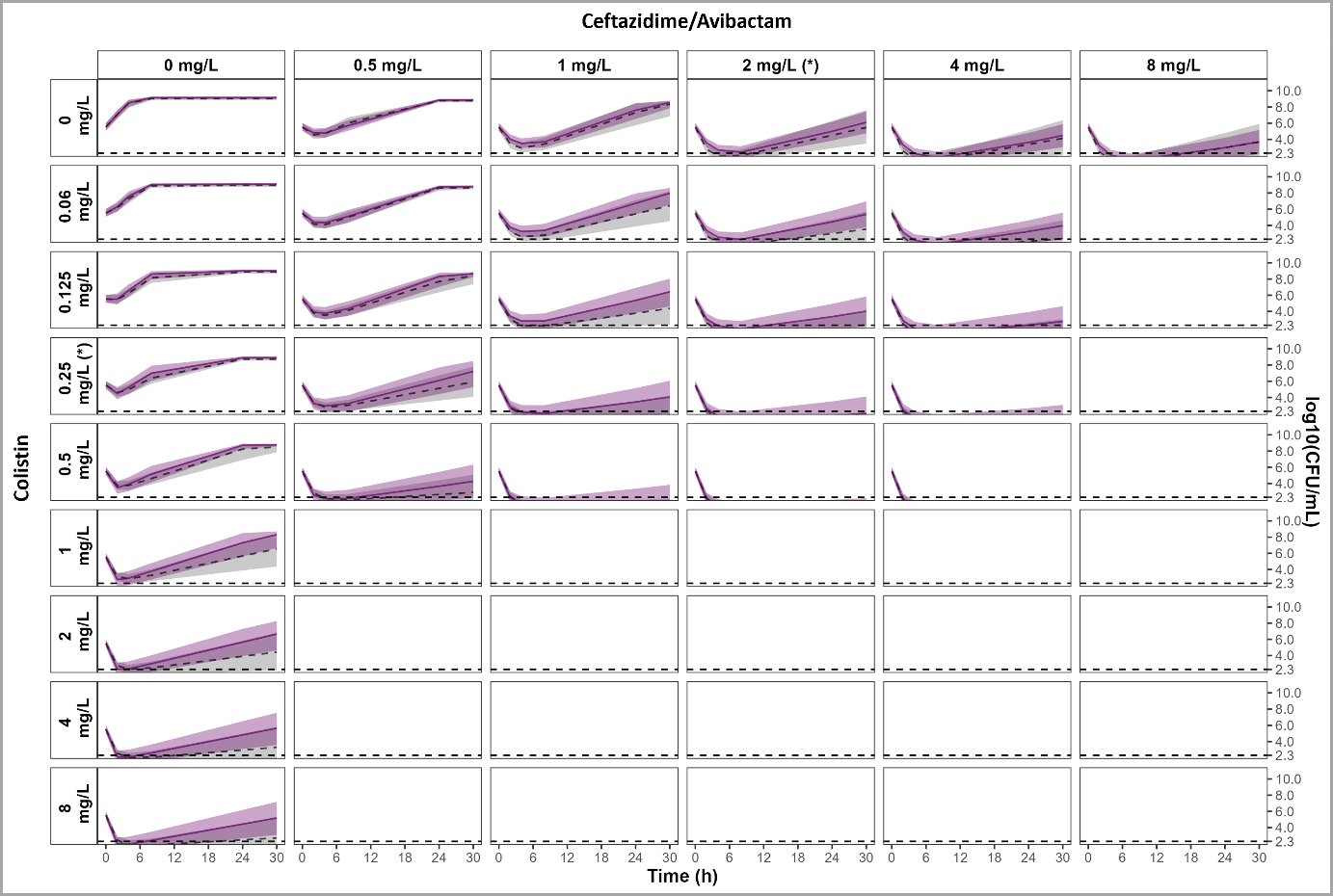
**

**Figure S6. Comparison of the 95%CIs of the effect predicted with the no IE (grey area) and with the IE (purple area) models at 5.10^5^ CFU/mL**

The 95%CI of the effect predicted with the no IE model, obtained by SIR, is represented by grey areas and the corresponding median percentile is represented by the dashed line. The 95%CI of the effect predicted with the IE model is represented by light purple areas and the median percentile is represented by the solid line. Both 95%CIs were systematically overlapping indicating that CFU counts over time were not significantly affected by the IE. The limit of quantification is represented by the horizontal dashed line at 2.3 log_10_CFU/mL. MICs of CZA and CST are indicated by (*).

**
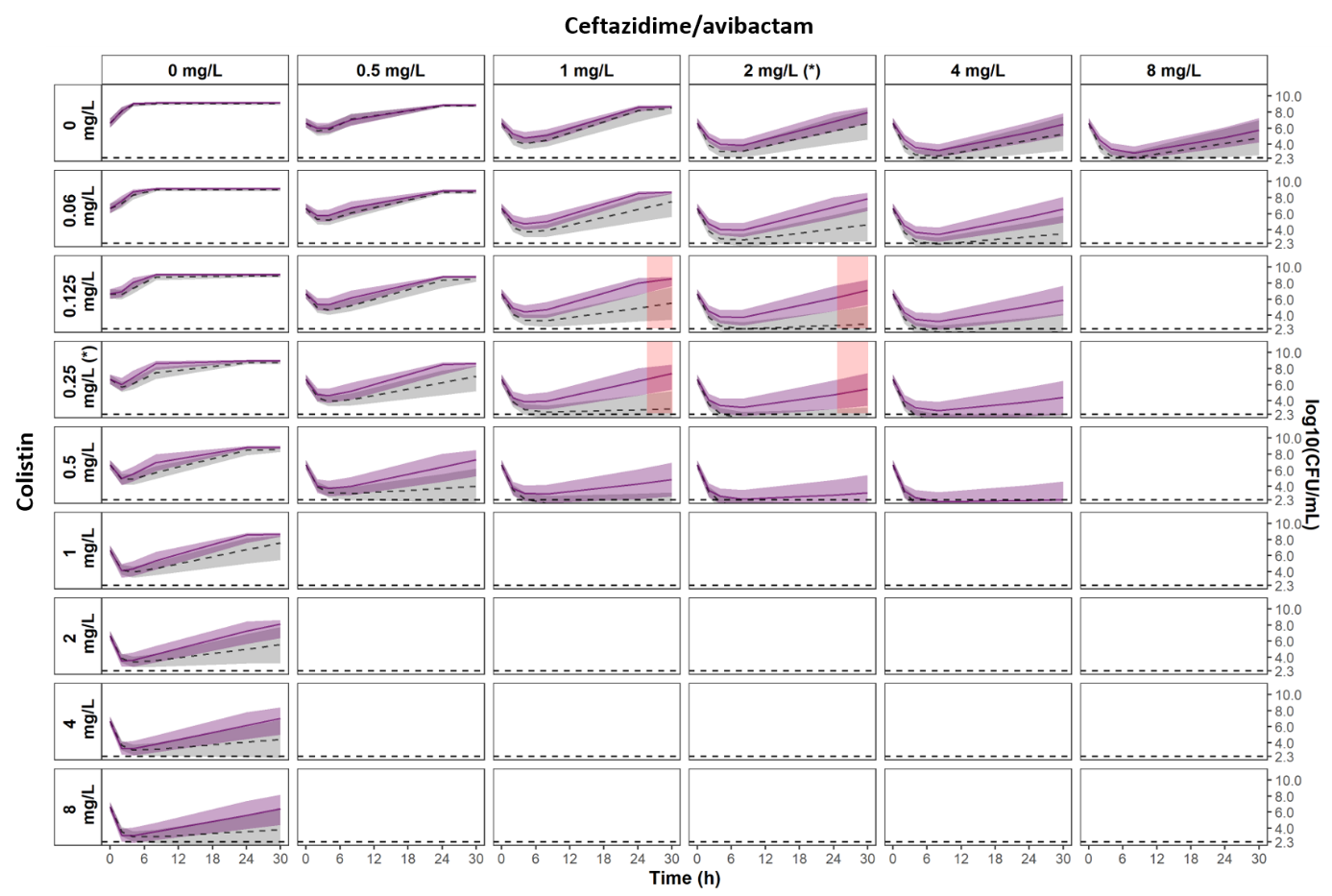
 Figure S7. Comparison of the 95%CIs of the effect predicted with the no IE (grey area) and with the IE (purple area) models at 10^7^ CFU/mL**

The 95%CI of the effect predicted with the no IE model, obtained by SIR, is represented by grey areas and the corresponding median percentile is represented by the dashed line. The 95%CI of the effect predicted with the IE model is represented by light purple areas and the median percentile is represented by the solid line. Statistically significant areas of decreased drug effect due to inoculum effect are highlighted in red (non-overlapping 95%CIs). The limit of quantification is represented by the horizontal dashed line at 2.3 log_10_CFU/mL. MICs of CZA and CST are indicated by (*).

**
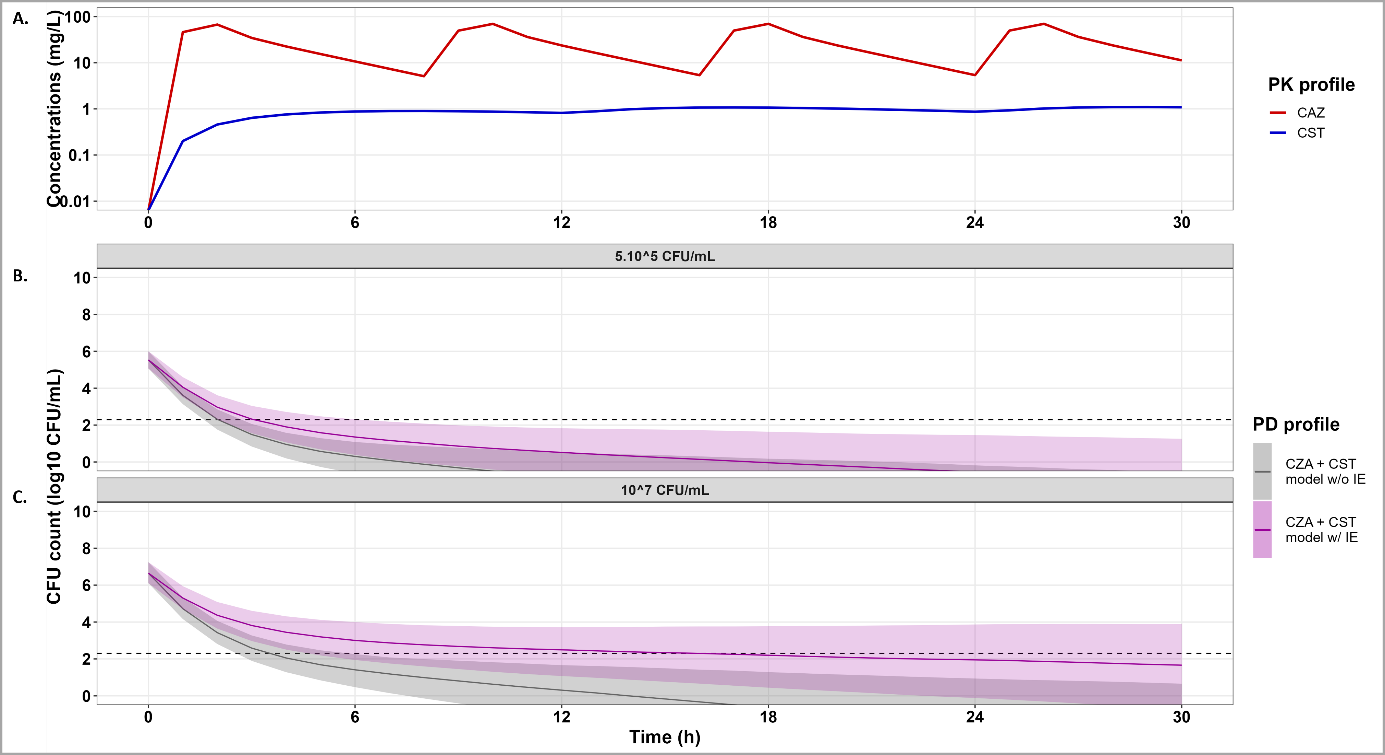
 Figure S8. PKPD simulation in combination, with the no IE (grey area) and with the IE (purple area) models, at 5.10^5^ CFU/mL (B.) and 10^7^ CFU/mL inoculum (C.) for typical free CAZ and CST concentrations (A.).**

Free PK profiles were simulated in typical patients after standard ceftazidime (2 g q8h as 2 h infusion) and colistin (9 MIU CMS + 4.5 MIU CMS q12h as 30 min infusion) doses. Statistically significant areas of decreased drug effect due to inoculum effect are highlighted in red (non-overlapping 95%CIs).

**Text S1. PKPD modelling**

*PKPD modelling of the no IE model*

In the first step of the analysis, single drug and combination effects at a 5.10^5^ CFU/mL standard inoculum were described by using a previously developed PKPD model (1), re-estimating the parameters on the basis of experimental data from the present study. This model is referred to as the “no IE model”. The difference with our previously published model is the addition of inter-experimental variability on the inoculum. The size of the inoculum as well as the inter-experimental variability were estimated for each inoculum size using solely CFU counts at T0. The bacterial growth was described using a logistic growth model with a single homogeneous bacterial population. Ceftazidime (with 4 mg/L of avibactam) and CST induced bacterial killing according to a sigmoidal Emax model and the bacterial regrowth observed in TKCs were modelled assuming empirical emergence of adaptive resistance under antibiotic exposure. The bacterial adaptation over time induced a reduction of the Emax parameter of the corresponding drug, proportionally to the fraction of adapted bacteria until reaching a residual effect when all bacteria reached the adapted state. The General PharmacoDynamic Interaction (GPDI) model (2), implemented under the Bliss Independence hypothesis, was used to characterize the PD interaction between CZA and CST.

*PKPD modelling of the IE model*

In the second step of the analysis, the no IE model was adapted to take into account the IE and fitted to the full dataset including all tested inocula. To describe the IE of each drug, multiple functions including linear, power and (sigmoidal) Emax equations were tested, resulting in a change of Emax, EC_50_ or Kon of the corresponding drug. In addition, the possibility that in combination each antibiotic modified the IE of the other drug was investigated using the GPDI model according to the following equation.

$${\gamma IE}_{X\_Combo}=\gamma{IE}_{X\_Mono}\times\left( 1 + \frac{{INT}_{IE} \times{ConcY}^{\gamma INT}}{{EC{50}_{INT}}_{Y}^{{\gamma INT}_{IE}} + {ConcY}^{\gamma INT}} \right)$$

Where, $\gamma{IE}_{X\_Combo}$ is the IE parameter of drug X in the presence of a concentration $ConcY$ of drug Y, $\gamma{IE}_{X\_Mono}$ is the IE parameter of drug X used alone (cf. Equation 1 in the main text), ${INT}_{IE}$ is the maximal fractional change of $\gamma{IE}_{X\_Mono}$ due to drug Y, $EC{{50}_{INT}}_{Y}$ is the concentration of drug Y required to reach 50% of ${INT}_{IE}$ and $\gamma INT_{IE}$ is the sigmoidicity parameter for this interaction.

The selection of the model that best described the observed data, so called “IE model”, was based on the difference in OFV between models.

*Comparison of the 95% confidence intervals of the IE model vs no IE model*

To assess the uncertainty of the model predictions and build the 95%CI of the no IE model, a Sampling Importance Resampling (SIR) procedure was applied with the SIR tool of PsN 5.3.0. (3). The covariance matrix estimated from the $COV step was used as proposal distribution. The number of samples was set to M = (1000, 1000, 1000, 2000, 2000) and the number of resamples was set to m = (200, 400, 600, 800, 1000). The SIR convergence was graphically assessed using the dOFV distribution diagnostic plot and additional iterations were added if the convergence was not reached at the end of the procedure. Subsequently, the individual predictions were simulated from the 1000 parameter vectors determined by SIR and summary statistics (median, 2.5 and 97.5 percentiles) of bacterial counts were calculated over time for each inoculum size in order to build the 95%CI of the no IE model.

The 95%CI of the IE model was built following the same procedure. The comparison of predicted effects over time according to whether experiments were performed with a single inoculum or with several inocula was performed by superimposing the 95%CIs predicted from the IE model and from the No IE model. If the 95%CI of the IE model was higher than the expected effect from the no IE model (*i.e.,* the lower boundary of the 95%CI of the IE model was above the upper limit of the 95%CI of the no IE model), a lower bacterial killing was observed and the drug effect was considered to be significantly reduced. If both 95%CIs overlapped, no difference could be highlighted and the difference was considered not significant. If the 95%CI of the IE model was lower than the expected effect from the no IE model (*i.e.,* the upper boundary of the 95%CI of the IE model was below the lower limit of the 95%CI of the no IE model), a significantly higher bacterial killing was observed. The comparison was performed over time for each inoculum size and at different drug concentrations.

References

1. Aubry R, Buyck J, Prouvensier L, Decousser J-W, Nordmann P, Wicha SG, Marchand S, Grégoire N. 2023. An improved PKPD modeling approach to characterize the pharmacodynamic interaction over time between ceftazidime/avibactam and colistin from in vitro time-kill experiments against multidrug-resistant Klebsiella pneumoniae isolates. Antimicrob Agents Chemother 67:e0030123.

2. Wicha SG, Chen C, Clewe O, Simonsson USH. 2017. A general pharmacodynamic interaction model identifies perpetrators and victims in drug interactions. Nat Commun 8.

3. SIR user guide. PsN 5.3.0. Revised 2019-05-08. https://uupharmacometrics.github.io/PsN/docs.html.

**Code S1. NONMEM control stream of the observed effect model with inoculum effect**

;; 1. Based on:

;; 2. Description: model w/ IE - full dataset

;; x1. Author: RAU

$PROBLEM In-vitro inoculum effect with the combination Ceftazidime/Avibactam + Colistin on K. pneumoniae N864

$INPUT ID CAZ_CONC CST_CONC TIME DV CMT BLQ INOC_TH INOC_OBS EXPID=DROP ID2 EVID

$DATA ../Data/260_CZA-CST_dataset_full_IEV.csv

IGNORE=I

$SUBROUTINES ADVAN13 TOL=9

$MODEL

COMP=(CAZ) ; Ceftazidime concentration (Avibactam fixed to 4 µg/mL)

COMP=(CST) ; Colistin concentration

COMP=(BTOT) ; Total bacteria

COMP=(ARON_CZA) ; Adapted fraction to CZA

COMP=(AROFF_CZA) ; Non adapted fraction to CZA

COMP=(ARON_CST) ; Adapted fraction to CST

COMP=(AROFF_CST) ; Non adapted fraction to CST

$PK

FL1=0

FL2=0

FL3=0

FL4=0

IF(INOC_TH.EQ.10000) FL1=1

IF(INOC_TH.EQ.500000) FL2=1

IF(INOC_TH.EQ.10000000) FL3=1

IF(INOC_TH.EQ.100000000) FL4=1

INOC_TV=THETA(1)*FL1 + THETA(2)*FL2 + THETA(3)*FL3 + THETA(4)*FL4 ; Estimated inoculum size typical value

INOC=INOC_TV*EXP(ETA(1)) ; Inter experimental variability around the typical inoculum size

KG=THETA(5) ; Bacterial growth rate constant (1/h)

BMAX=THETA(6) ; Bacterial count in the stationary phase (log10 (CFU/mL))

EMAX_CZA=THETA(7) ; Maximum killing rate of CZA effect (h-1)

EC50_CZA=THETA(8) ; CZA concentration needed to reach 50% of EMAX (mg/L)

GAMMA_CZA=THETA(9) ; Hill's factor

KON_CZA=THETA(10) ; Rate of adaptation development to CZA(1/h)

KOFF=THETA(11) ; Rate of adaptation reversal (l/h) fixed to 0

AR_CZA=THETA(12) ; Effect of adapated bacteria on drug effect

GAMMA_IE_CZA=THETA(13) ; Power parameter for inoculum effect model

EMAX_CST=THETA(14) ; Maximum killing rate of CST effect (h-1)

EC50_CST=THETA(15) ; CST concentration needed to reach 50% of EMAX (mg/L)

GAMMA_CST=THETA(16) ; Hill's factor

KON_CST=THETA(17) ; Rate of adaptation development to CST(1/h)

AR_CST=THETA(18) ; Effect of adapated bacteria on drug effect

GAMMA_IE_CST=THETA(19) ; Power parameter for inoculum effect model

INT_EMAX_CZA=THETA(20) ; Interaction parameter = maximal fractional change of EMAX_CZA due to CST

EC50_INT_EMAX=THETA(21) ; CST concentration to reach half of INT_EMAX_CZA (mg/L)

GAMMA_INT_EMAX=THETA(22) ; Sigmoidicity parameter of the interaction relationship

; Initial conditions

IF(A_0FLG.EQ.1) THEN

A_0(1)=CAZ_CONC

A_0(2)=CST_CONC

A_0(3)=10**INOC

A_0(4)=0

A_0(5)=1

A_0(6)=0

A_0(7)=1

ENDIF

$DES

; Bacterial growth

BTOT=A(3)

PLATEAU=1-(BTOT/(10**BMAX))

; Inoculum effect on EMAX_CZA - power model

EMAX_CZA_INOC=EMAX_CZA*(INOC/LOG10(500000))**GAMMA_IE_CZA

; Inoculum effect on EC50_CST - power model

EC50_CST_INOC=EC50_CST*(INOC/LOG10(500000))**GAMMA_IE_CST

; Adaptation to Ceftazidime/Avibactam

ADAPT_CZA=0

IF(A(1).GT.0) ADAPT_CZA=KON_CZA

E_ADAPT_CZA=AR_CZA*A(4)

; Adaptation to Colistin

ADAPT_CST=0

IF(A(2).GT.0) ADAPT_CST=KON_CST

E_ADAPT_CST=AR_CST*A(6)

; PD interaction between CST & CZA - GPDI model with CST modifying EMAX_CZA

INTERACTION=0

IF(A(1).GT.0.AND.A(2).GT.0) INTERACTION=(INT_EMAX_CZA*A(2)**GAMMA_INT_EMAX)/(EC50_INT_EMAX**GAMMA_INT_EMAX+A(2)**GAMMA_INT_EMAX)

; Ceftazidime/Avibactam drug effect

KILL_CZA=0

IF(A(1).GT.0) KILL_CZA=(EMAX_CZA_INOC*(1-E_ADAPT_CZA)*(1+INTERACTION)*A(1)**GAMMA_CZA)/(EC50_CZA**GAMMA_CZA+A(1)**GAMMA_CZA)

; Colistin drug effect

KILL_CST=0

IF(A(2).GT.0) KILL_CST=(EMAX_CST*(1-E_ADAPT_CST)*A(2)**GAMMA_CST)/(EC50_CST_INOC**GAMMA_CST+A(2)**GAMMA_CST)

; Bliss Independence

IF(EMAX_CZA.GT.EMAX_CST) EMAX=EMAX_CZA

IF(EMAX_CZA.LT.EMAX_CST) EMAX=EMAX_CST

E_COMB=((KILL_CZA/EMAX)+(KILL_CST/EMAX)-((KILL_CZA/EMAX)*(KILL_CST/EMAX)))*EMAX

; ODE

DADT(1)=0 ; Ceftazidime concentration constant over time

DADT(2)=0 ; Colistin concentration constant over time

DADT(3)=(KG*PLATEAU-E_COMB)*A(3) ; Total bacteria over time

DADT(4)=ADAPT_CZA*A(5)-KOFF*A(4) ; Adapted fraction to CZA over time

DADT(5)=KOFF*A(4)-ADAPT_CZA*A(5) ; Non adapted fraction to CZA over time

DADT(6)=ADAPT_CST*A(7)-KOFF*A(6) ; Adapted fraction to CST over time

DADT(7)=KOFF*A(6)-ADAPT_CST*A(7) ; Non adapted fraction to CST over time

$THETA

(3.85) FIX ; LGINOC_4

(5.54) FIX ; LGINOC_5.7

(6.65) FIX ; LGINOC_7

(7.76) FIX ; LGINOC_8

(1.64) ; KG

(9.13) ; BMAX

(5.88) ; EMAX_CZA

(0.422) ; EC50_CZA

(1) ; GAMMA_CZA

(0.326) ; KON_CZA

(0) FIX ; KOFF

(0.754) ; AR_CZA

(-0.267) ; GAMMA_IE_CZA

(13.9) ; EMAX_CST

(0.283) ; EC50_CST

(1.17) ; GAMMA_CST

(1.24) ; KON_CST

(0.901) ; AR_CST

(1.66) ; GAMMA_IE_CST

(-0.546) ; INT_EMAX_CZA

(0.3) FIX ; EC50_INT_EMAX

(1) FIX ; GAMMA_INT_EMAX

$SIGMA 1.1 ; SIGMA

$OMEGA 0.00188 FIX ; IEV_INOC

$ERROR

A1=A(1)

A2=A(2)

A3=A(3)

A4=A(4)

A5=A(5)

A6=A(6)

A7=A(7)

BTOT_ERR = A3

IF(BTOT_ERR<1e-6) BTOT_ERR=1e-6

;Sim_start

IF(CMT.EQ.3.AND.BLQ.EQ.1) THEN ;M3 method for data BLQ

IPRED = LOG10(BTOT_ERR)

W= SQRT(SIGMA(1))

IRES = -9999

IWRES = -9999

F_FLAG=1

MDVRES=1

LOQ=LOG10(200) ;; LOQ = 200 CFU/mL

DUM=(LOQ-IPRED)/(W+0.00001)

CUMD=PHI(DUM) ;; PHI = cumulative density function

Y = CUMD

ENDIF

IF(CMT.EQ.3.AND.BLQ.EQ.0) THEN

IPRED = LOG10(BTOT_ERR)

W= SQRT(SIGMA(1))

IRES = DV-IPRED

IWRES = (IRES/(W+0.00001))

;Sim_start

F_FLAG=0

MDVRES=0

;Sim_end

ERR1= EPS(1)

Y= IPRED+ERR1

ENDIF

$ESTIMATION METHOD=1 INTERACTION LAPLACIAN MAXEVAL=9999 SIGDIG=3 SIGL=9 SIGLO=9 PRINT=5 NOABORT POSTHOC

$COVARIANCE PRINT=E UNCONDITIONAL

$TABLE ID ID2 TIME EVID INOC_TH INOC CAZ_CONC CST_CONC DV CMT BLQ A1 A2 A3 A4 A5 A6 A7 DV PRED IPRED RES IRES WRES IWRES ONEHEADER NOPRINT FILE=sdtab46

$TABLE ID ID2 TIME EVID INOC_TH INOC A1 A4 A5 KILL_CZA ADAPT_CZA E_ADAPT_CZA EMAX_CZA_INOC A2 A6 A7 KILL_CST ADAPT_CST E_ADAPT_CST EC50_CST_INOC A3 DV PRED RES WRES IPRED IRES IWRES ONEHEADER NOPRINT NOAPPEND FILE=patab46.csv FORMAT=,F12.2
